# Genito-Urinary Surgery and Venereal Diseases

**Published:** 1899-06

**Authors:** 


**Genito-Urinary Surgery and Venereal Diseases.** By J. WILLIAM WHITE, M.D., and EDWARD MARTIN, M.D. Pp. xix., 1061. Philadelphia: J. B. Lippincott Company. 1898.

The need is often felt of a book which gives more information than an ordinary text-book, and discusses more fully various methods of treatment. Such a need, so far as genito-urinary surgery and venereal diseases are concerned, will be supplied in this book. It is a work of much value, written by two well-known American surgeons. Indeed, Professor White is perhaps as well known in this country in connection with the treatment of hypertrophied prostate by castration as any American surgeon.

The anatomy of the various genito-urinary organs is given before their injuries and diseases are described; and this we are inclined to think a very useful and convenient arrangement. As an illustration of how fully the subjects are considered, we may refer to a table giving the differential diagnosis of the various parts of the urethra affected in gonorrhœa. The attempt to give the reader an idea of the appearance of gonorrhœal conjunctivitis by means of an uncoloured photograph, we must say, is a complete failure. The coloured photographs of venereal sores and syphilitic skin eruptions, also completely fail to represent the disease. Even coloured plates of venereal sores give a very imperfect idea of them. But the other illustrations in the book are very good, and some pictures of hypertrophied prostates from Watson are particularly fine.

No less than 240 pages are devoted to syphilis. We are glad to see syphilitic epididymitis mentioned, though it is not very clearly described; but attention is called to the similarity of this disease to tuberculous conditions, a subject seldom referred to in books. We are interested in the strong view expressed in favour of litholapaxy in boys, and the encouraging statistics which are given. The important subject of cystoscopy receives adequate consideration, and some very needful cautions are given as to the mistakes which may be made in the interpretation of the appearances presented. We notice a reproduction of a very clear skiagram of a stone in the kidney, and consider that the subject of renal surgery receives careful and comprehensive treatment. Diseases of the seminal vesicles, about which our knowledge is getting more precise, are here well described, and it is perhaps needless to say that we can hardly expect a better account of the treatment of prostatic hypertrophy by castration and vasectomy than we have in this volume, no doubt from the pen of Professor White. In the treatment of rupture of the urethra we are not told to cut down on the rupture if we can get a catheter into the bladder, nor is any mention made of the fact that some eminent surgeons now recommend this proceeding in all cases in which the laceration is at all extensive, as a safeguard against urinary infiltration. But various views are as a rule very fully given, and in the pages devoted to the treatment of extroversion of the bladder the different methods are instructively discussed.

We strongly recommend the book as a modern guide to this branch of surgery.

